# Pulmonary Embolism as an Initial Presentation of Adrenocortical Carcinoma

**DOI:** 10.14740/wjon814w

**Published:** 2014-06-25

**Authors:** Soe Yu Aung, Phillip Parente, Joseph McKendrick

**Affiliations:** a Box Hill Hospital, Eastern Health Victoria, Nelson Road, Box Hill, VIC 3128, Australia

**Keywords:** Adrenocortical carcinoma, Endocrine tumor, Pulmonary embolism

## Abstract

Adrenocortical carcinomas (ACCs) are rare and often aggressive with more than 50% of the cases already in stage III-IV (ENSAT) at the time of diagnosis. Nearly 60% of ACCs present with hormone overproduction syndromes (Cushing’s syndrome and/or virilization), while the rest present with abdominal mass or incidental finding. Aggressive surgical resection is the mainstay of treatment usually followed by adjuvant mitotane monotherapy. For the advanced stage, adjuvant radiotherapy and combined chemotherapy with mitotane therapy can be added for survival benefit. Here, we would like to report a case of stage III high-grade ACC without syndromes of hormone overproduction, initially presented with pulmonary embolism. It was rapidly progressive with metastases to lungs, peritoneum and bone despite aggressive surgery followed by adjuvant mitotane monotherapy. However, after palliative radiotherapy to thoraco-lumbar spine for spinal cord compression, and adding chemotherapy (six cycles of EDP: etoposide, doxorubicin, cisplatin) to mitotane, a significant partial remission was achieved. He has had 24 months of progression-free survival, and is currently on mitotane monotherapy with cortisol replacement. Discussion will support multimodality therapy for stage III high-grade ACC with surgery immediately followed by adjuvant radiotherapy and combined chemotherapy with mitotane therapy to prevent local recurrence and distant metastases.

## Introduction

The incidence of adrenocortical carcinomas (ACCs) is very low with 1 - 2 per million population per year [1], and female to male ratio of 2:1 [2]. It has bimodal age distribution with the peaks before the age of 5 and in the fourth and fifth decade of life [1]. About 60% of ACCs are functional with overproduction of glucocorticoids (Cushing’s syndrome) and/or androgens (virilization) [1, 2], and the remaining ACCs are non-functional, presenting as abdominal mass related symptoms, constitutional symptoms or incidental finding. On initial presentation, about 50% of adult ACCs are already in advanced stages [3]. ACCs are usually aggressive in nature, and the usual metastatic sites are lungs (71%), lymph node (68%), liver (42%) and bone (26%) [4]. Prognosis of ACCs is poor with 5-year disease-specific survival rate of 13% in distant metastatic disease, and 50-82% in disease without distant spread [3].

Even though the mainstay of treatment for ACCs is aggressive surgical resection followed by adjuvant mitotane therapy, there is limited evidence of the benefits from adjuvant radiotherapy and combined chemotherapy with mitotane therapy.

Here, we would like to report a case of stage III high-grade ACC with capsular invasion in a 38-year-old male patient, who presented with pulmonary embolism and left flank mass, without syndromes of hormone overproduction.

## Case Report

**Figure 1:**
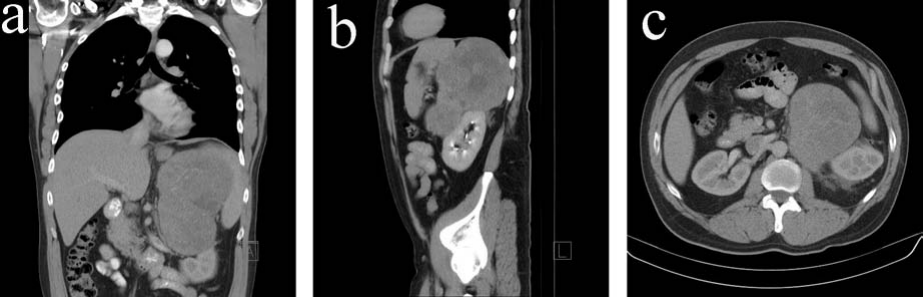
(a) CT scan (coronal view) on initial diagnosis. (b) CT scan (sagittal view) on initial diagnosis. (c) CT scan (transverse view) on initial diagnosis.

A 38-year-old man with no significant past medical history presented with left-sided chest pain, dyspnoea on exertion and left flank pain in April 2011. He was found to have left lower lobe segmental pulmonary embolus on a VQ scan, together with an incidental finding of a large soft tissue mass (15.5 × 12.0 × 16.7 cm) with no arterial phase enhancement, immediately superior to the left kidney in the CT scan. No local or distant metastases were noted in the staging CT scan (Fig. 1).

He underwent an open laparotomy for removal of the tumor, left kidney and spleen. Pulmonary embolism was treated with therapeutic enoxaparin.

**Figure 2:**
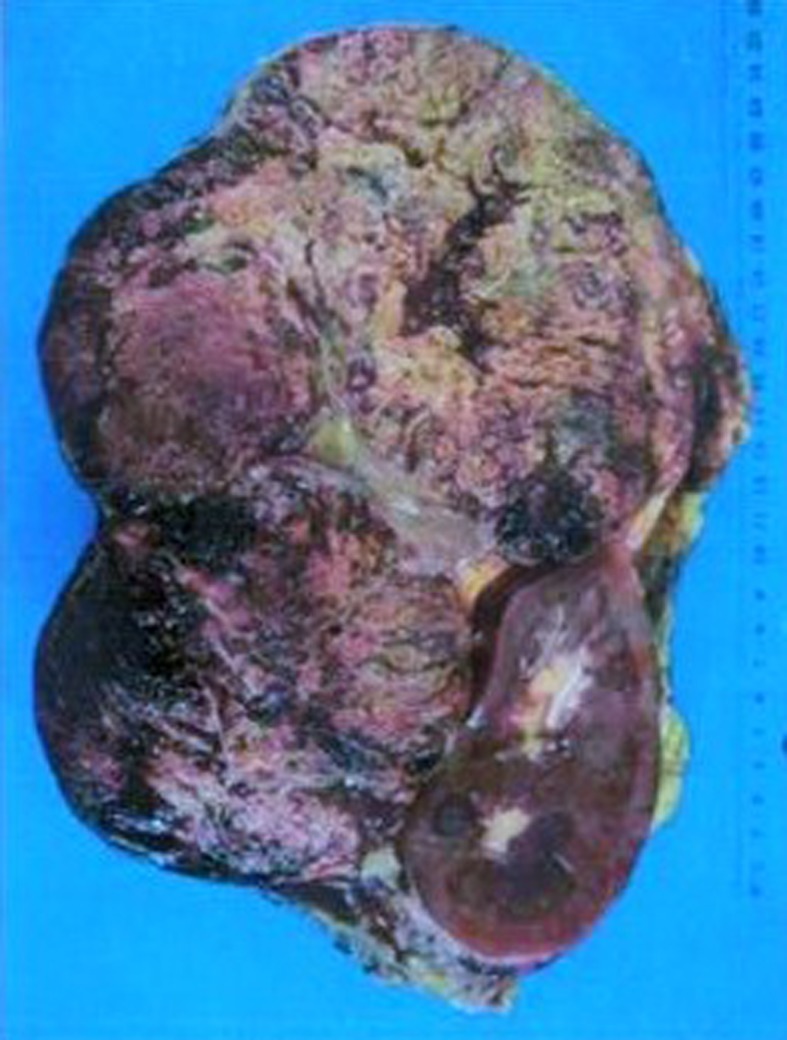
Macroscopic appearance of the resected tumor.

Macroscopically, a large, well circumscribed tumor (210 × 160 × 100 mm) with soft granular tan cut surfaces was found to be displacing the left kidney posteriorly and inferiorly (Fig. 2). No adrenal gland was identified in the specimen or within the tumor. A fibrous pseudo-capsule separated the tumor from the planes of resection. Tumor was separated from the kidney by renal capsule, and found to be pressing on the renal vein without extension into it. The spleen and the left kidney were of normal appearance.

**Figure 3:**
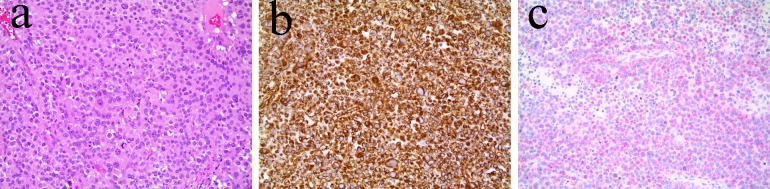
(a) Hematoxylin and eosin stain. (b) Vimentin stain. (c) Melan-A stain.

Microscopically, the tumor completely replaced the left adrenal gland, and was composed of sheets of large pleomorphic cells with eosinophilic cytoplasm (Fig. 3a). It demonstrated all the risk factors for development of metastatic disease with diffused growth pattern, vascular invasion into small venules but not into the renal vein, extensive tumor necrosis, foci of coarse stromal calcification, > 10 mitoses per 100 high power fields, cellular pleomorphism and capsular invasion. There was no evidence of malignancy in one left renal hilar lymph node and one splenic lymph node.

Tumor cells were strongly positive with Vimentin (Fig. 3b), weakly positive with Melan-A (Fig. 3c), and were negative with AE1/AE3 and S100.

The above findings classified the tumor as stage III (T3N0M0) undifferentiated large cell ACC (as per both AJCC and ENSAT staging system).

Since the initial presentation there were no signs and symptoms of hypercortisolism, though urine androsterone, etiocholanolone, tetrahydrocortisol and tetrahydrocortisone level were high. The latter went back to normal after the radical surgery.

**Figure 4:**
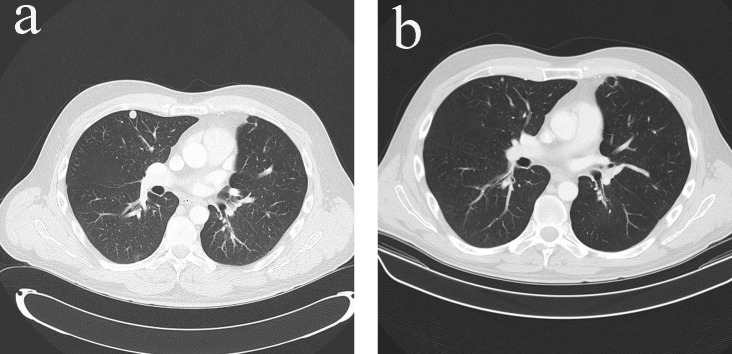
(a) Pulmonary metastases in CT scan (transverse view) before additional chemotherapy with EDP. (b) Resolution of pulmonary metastases in CT scan (transverse view) after additional chemotherapy with EDP.

Adjuvant mitotane monotherapy (3 - 6 g/day) together with cortisol replacement was initiated after the surgery. The patient enjoyed disease-free period of 7 months until he developed metastatic disease in bones, lungs and peritoneum (Fig. 4a, 5a). At time of presentation of relapse he was complaining of thoracic back pain which precipitated MRI spine which showed T12 cord compression requiring palliative radiotherapy (30 Gy in 10 fractions). Six cycles of etoposide, doxorubicin and cisplatin (EDP) were added to mitotane over a period of 7 months, resulting in excellent partial response to treatment, i.e. complete response of all peritoneal nodules, near complete response of the multiple pulmonary nodules except one sub-centimeter nodule in each lung and stable bony metastases (Fig. 4b, 5b). The patient is now on mitotane monotherapy only (3 - 6 g/day) with stable disease. Therapeutic enoxaparin was ceased after 27 months of treatment for pulmonary embolism with no further thromboembolic complications.

**Figure 5:**
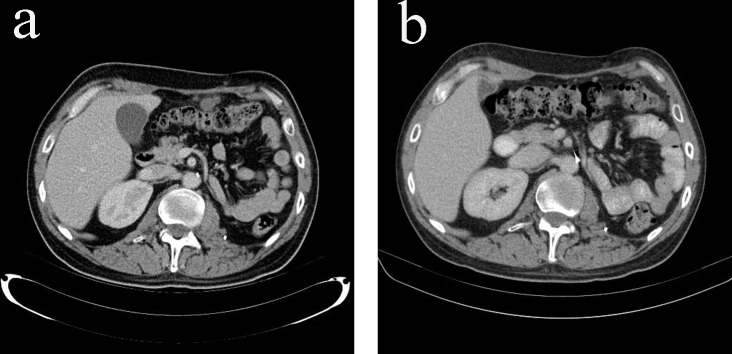
(a) Peritoneal metastases in CT scan (transverse view) before additional chemotherapy with EDP. (b) Resolution of peritoneal metastases in CT scan (transverse view) after additional chemotherapy with EDP.

## Discussion

Majority of the adult ACCs (60%) have syndromes of hormone overproduction on initial presentation, and the remaining are non-functional, presenting with an abdominal mass, constitutional symptoms or an incidental finding. Rarely, ACCs can present with tumor extending into the inferior vena cava and even into the right atrium [5]. A case of ACC with clinical feature of pheochromocytoma was also reported [6]. The initial presentation in our case was pulmonary embolism, and the ACC was the incidental finding.

The only potentially curative treatment for ACCs is complete surgical resection [7]. For resectable stage I to III ACCs, adjuvant mitotane (an adrenocorticolytic drug) therapy was shown to improve recurrence-free survival and median overall survival duration [8]; however, there is no data available yet regarding benefit of adjuvant chemotherapy with or without mitotane. Even though there is no adequate data on the role of adjuvant radiotherapy, to reduce the risk of local recurrence which is highest in the first 2 years, German ACC registry recommends adjuvant radiotherapy be started no later than 3 months after surgery for all patients with microscopically incomplete (R1 or R2) or uncertain (Rx) margin status, and for those with stage III disease (ENSAT criteria) [7].

For recurrent or metastatic ACCs (stage IV, ENSAT criteria), surgical resection can also prolong survival in surgically accessible cases [9]. Chemotherapy plus mitotane has better benefits than mitotane monotherapy in advanced ACCs. EDP plus mitotane is shown to have higher rates of tumor response and longer median progression-free survival than steptozotocin plus mitotane in the first international randomized trial in locally advanced and metastatic adrenocortical carcinoma treatment (FIRM-ACT) trial [10].

In our patient, initially, he presented with stage III left-sided ACC (ENSAT), and underwent aggressive surgical resection including the tumor, spleen and the left kidney. Subsequently, adjuvant mitotane therapy was started with cortisol replacement. However, he did not have adjuvant radiotherapy. Seven months after the diagnosis, while he was on adjuvant mitotane monotherapy, the patient developed multiple pulmonary, peritoneal and bony metastases without hypercortisolism. After going through palliative radiotherapy to the thoroco-lumbar spine (35 Gy in 10 fractions) for spinal cord compression, chemotherapy (six cycles of EDP) was added to the adjuvant mitotane therapy. As a result, peritoneal metastases resolved completely with only two stable sub-centimeter lung nodules remained and stable bony metastatic disease. He has been followed up 3-monthly till now with no evidence of disease progression leading to progression-free survival of 24 months with continuous mitotane monotherapy.

In conclusion, ACCs can have different unusual clinical presentations; pulmonary embolism is the initial clinical presentation in our case. The experience in our case supports multimodality therapy for stage III high-grade ACC due to the short disease free interval from surgical resection and relapse with metastatic disease. Multimodality therapy includes adjuvant radiotherapy and EDP in addition to mainstay aggressive surgical resection and adjuvant mitotane therapy.

## Abbreviations

ACC: adrenocortical carcinoma
AJCC: American Joint Committee on Cancer
ENSAT: The European Network for the Study of Adrenal Tumors
EDP: etoposide, doxorubicin, and cisplatin
